# Effect of Food and Vitamin D Supplements on the Serum 25(OH)D_3_ Concentration in Children during Winter Months

**DOI:** 10.3390/foods3040632

**Published:** 2014-12-11

**Authors:** Ellen van der Gaag, Lisanne Brekhoff

**Affiliations:** 1 Hospital Group Twente, Geerdinksweg 141, 7555 DL Hengelo, The Netherlands; 2 Medical School University of Groningen, Hanzeplein 1, Groningen, The Netherlands; E-Mail: l.brekhoff@student.rug.nl

**Keywords:** children, dietary vitamin D, nutrients, vitamin D deficiency, vitamin D supplements

## Abstract

Aim: To determine the contribution of food and vitamin D supplements on the serum 25-hydroxyvitamin D_3_ (25(OH)D_3_) concentration between October and April in a northern country (almost absent vitamin D synthesis by sunlight). Methods: Children aged 1–18 years were selected who visited the general pediatrician with a complaint whereby serum 25(OH)D_3_ concentration was determined. The intake of vitamin D was calculated based on a dietary questionnaire. Results: 51.1% of the 174 children had a serum 25(OH)D_3_ concentration below 50 nmol/L, 9.2% had a serum 25(OH)D_3_ concentration below 30 nmol/L. Adolescents showed lower concentrations compared to younger children. There was a positive correlation between the total amount of vitamin D obtained from food and the serum 25(OH)D_3_ concentration (*r* = 0.218, *p* = 0.004). The intake of milk contributed more to the serum 25(OH)D_3_ concentration compared to the intake of artificial supplementation, butter or fish. Conclusions: In the absence of vitamin D synthesis by sunlight, vitamin D obtained from food has a significant influence on the serum 25(OH)D_3_ concentration in children. Vitamin D supplements can be described as trivial. This means we should pay more attention to food as a natural source of vitamin D.

## 1. Introduction

The main source of vitamin D_3_ for people is exposure to sunlight [1,2]. In northern countries however, such as The Netherlands, the skin only synthesizes vitamin D_3_ between April and October [1,3]. During the colder months, there is much less vitamin D_3_ synthesis in the skin because of the latitude and due to sun rays entering the atmosphere at a more oblique angle. Therefore, ultraviolet-B (UVB) photons have to pass through the ozone at a greater distance and more UVB photons are efficiently absorbed by ozone [4].

Less than 10% of vitamin D is obtained from dietary sources and vitamin D supplements [4]. The major foods that naturally contain vitamin D_3_ are (wild caught) salmon, oily fish, cod liver oil and dairy products. Some foods are fortified with vitamin D, but in Europe there are many countries that do not have standard fortification of food groups [1,4,5,6]. In The Netherlands the foods fortified with vitamin D include margarine, low fat margarine, baby food and baby/grow up milk, but no natural milk [7,8].

In The Netherlands, the recommended serum 25-hydroxyvitamin D_3_ (25(OH)D_3_) concentration for children is greater than 30 nmol/L [6]. In other continents or countries, such as The United States, Germany, Austria and Switzerland, the recommended serum 25(OH)D_3_ concentration is above 50 nmol/L [4,6].

There is limited data available on vitamin D deficiency (VDD) among the European pediatric population [9]. At the end of the winter, the prevalence of VDD is at its highest because the summer supplies are utilized [1,3]. Weggemans *et al.* [3] reported that in The Netherlands, 5% of the light-skinned children and 15%–30% of the dark-skinned children have VDD at the end of winter.

Because of the importance of vitamin D for many functions of human body and due to the limited vitamin D synthesis in the skin between October and April, it is important to have another source of vitamin D during these months.

Since vitamin D is also obtained from dietary sources and vitamin D supplements, this study evaluated the effects of the intake of food and vitamin D supplements on the serum 25(OH)D_3_ concentration in children in The Netherlands between October and April.

## 2. Experimental Section 

### 2.1. Study Design

The study was an epidemiologic pilot study conducted at the ZGT Hengelo, The Netherlands, covering the period from February 2011 to April 2013 (with exclusion of the months from May to September).

### 2.2. Study Population

Children aged 1–18 years were selected who visited the general pediatrician with general pediatric complaints (like tiredness, abdominal pain, respiratory infections) whereby serum 25(OH)D_3_ concentration was determined. They were referred by a general practitioner.

### 2.3. Measurements

A custom-made isotope dilution LC-MS/MS method is used for the analysis of vitamin D (25-OH-vitamin D_3_). The method starts with protein precipitation of the serum proteins with acetonitril containing deuterated internal standard (D_6_-25-OH vitamin D_3_). This step also releases vitamin D from its binding proteins. After centrifugation and removing and evaporating the supernatant, the sample is reconstituted in 70% methanol followed by a centrifugation step. Reversed phase (C8) chromatographic separation was performed on a Shimadzu HPLC system ('s-Hertogenbosch, The Netherlands), consisting of two LC 20AD pumps, a SIL-20AC autosampler, and a CTO 20A column oven. Mass spectrometric detection was carried out on a Q-Trap 3200 mass spectrometer from Applied Biosystems (Foster City, CA, USA), in the MRM (multiple reaction monitoring) mode using APCI (atmospheric pressure chemical ionization) as an ionization method. At two concentrations (42 and 206 nmol/L) the intra-assay CVs (coefficients of variation) were calculated to be 3.6% and 2.7%, respectively and the inter-assay CVs 4.0% and 3.3%, respectively. The method is linear up to 1500 nmol/L and has a limit of quantification of 3 nmol/L. This is an excellent alignment with NIST (SRM 972) standards. Comparison to other laboratories was continuously observed by participating in the DEQAS (The Vitamin D External Quality Assessment Scheme) proficiency testing.

Information was collected on the vitamin D intake with use of a food questionnaire. The investigator noted the kind and amount of food the children had eaten during the past week. The mean was calculated to estimate the daily intake. To circumvent investigator bias the same investigator was used to take the food questionnaires for all the children. The intake was noted of the type and quantity of milk in milliliters, the type of butter the children put on their bread, the number of slices of bread that was buttered, the type and quantity of fish eaten and the use of vitamin D supplements. The medical complaints that brought them to the doctor was obtained, the amount of time the child spent playing outdoors every day, and the month of the year in which the serum 25(OH)D_3_ concentration was determined. The amount of vitamin D per day obtained from food and vitamin D supplements was calculated. For example, to calculate the vitamin D content from fish, the parents were asked to specify the amount and type of fish the child ate that week. Since there are big differences in the amount of vitamin D between oily fish and other types of fish, the Nederlands Voedingsstoffenbestand (NEVO) tables [10] were consulted to calculate the actual individual intake.

When a food was missing in the NEVO table, the super market was visited and food labels were read to determine the amount of vitamin D in the food. Each portion of butter the children put on one slice of bread was assumed to be 5 g, and one portion of fish was assumed to be 55 g.

### 2.4. Statistical Analysis

Statistical analysis was carried out using IBM SPSS Statistics 20. For normally-distributed variables, means and standard deviations were calculated and for non-normal distributions, medians and interquartile ranges (IQR) were calculated. A *p* value of less than 0.05 was considered significant.

Linear regression analysis was used to determine the specific effect of the age of the child on the total amount of vitamin D from food. In other words, could the age of the child be a factor of any influence on the 25(OH)D_3_ concentration. It was also used to determine the separate effect of the variables of gender, and the total amount of vitamin D obtained from food, milk, butter, fish and vitamin D supplements on the serum 25(OH)D_3_ concentration. The correlation coefficients were calculated using the Pearson test. Thereafter, multiple regression analysis was used to determine the effect of all the significant variables on the serum 25(OH)D_3_ concentration.

To determine the effect of different types of milk on the serum 25(OH)D_3_ concentration, the children were classified into five groups: children who did not drink milk, children who drank yogurt drinks and chocolate milk, children who drank semi-skimmed milk, children who drank whole milk, and the residual group. To investigate the influence of different types of butter on the serum 25(OH)D_3_ concentration, the children were classified into four groups: children who did not use butter, children who used low fat margarine, children who used margarine and children who used dairy butter. To determine the effect of the different types of milk and butter on the serum 25(OH)D_3_ concentration, dummy variables were made and linear regression analysis was carried out. Subsequently, correlation coefficients were calculated using the Pearson test and thereafter multiple regression analysis was used to determine the effect of all the significant variables on the serum 25(OH)D_3_ concentration.

### 2.5. Ethical Committee

No intervention was given. Existing laboratory results from standard patient care were used and therefore ethical consideration by an ethics committee was not necessary. For the use of the questionnaires, all patients agreed to participate in the collection of the data. The data was recorded and subsequently coded anonymously by the investigator.

## 3. Results and Discussion

### 3.1. Descriptives

There were 174 participating children and the median age was 8.5 years (IQR, 5.0–13.1). Of these children, 51.1% were female (Table 1). The medical complaints differed per child. 16.1% of the children were referred for abdominal pain, 18.4% suffered from tiredness, 6.9% had bronchial hyper reactivity and 5.7% had recurrent respiratory tract infections. Other problems were constipation, obesity, diarrhea, dizziness or asthma.

**Table 1:** Baseline characteristics.

Characteristic	Patients ( *n* = 174)
Age, median (IQR) (years)	8.5 (5.0–13.1)
Gender, number (%)	
Female	89 (51.1%)
Male	85 (48.9%)
Serum 25(OH)D concentration, mean ± SD (nmol/L)	52.0 ± 18.4
Vitamine D obtained with the total amount of food per day, mean ± SD (μg)	1.3 *±* 1.9
Vitamin D obtained with milk per day, mean ± SD (μg)	0.5 *±* 1.8
Vitamin D obtained with butter per day, mean ± SD (μg)	0.7 *±* 0.5
Vitamin D obtained with fish, mean ± SD (μg)	0.1 *±* 0.2
Vitamin D obtained with vitamin D supplements, mean ± SD (μg)	3.4 *±* 4.8

IQR = Interquartile range; SD = standard deviation.

**Figure 1:**
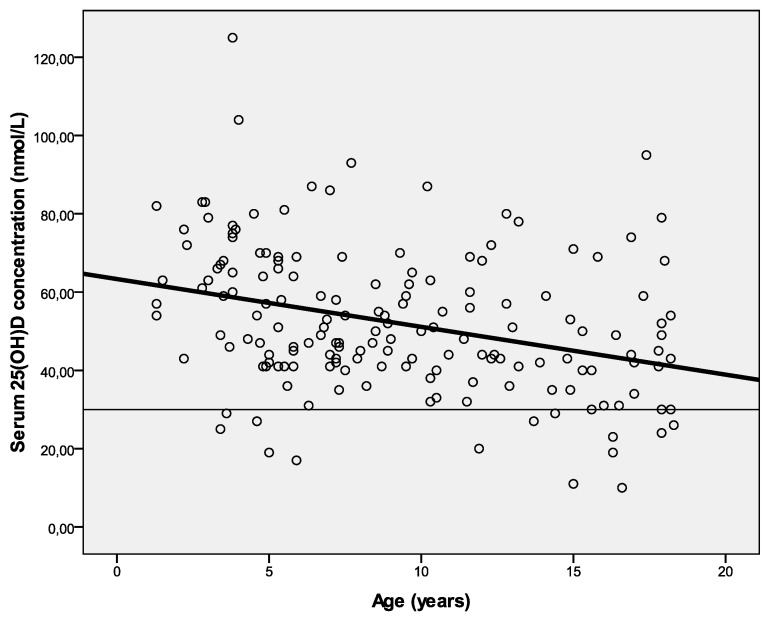
Mean serum 25(OH)D_3_ concentration per age.

The mean serum 25(OH)D_3_ concentration was 52.0 ± 18.4 nmol/L (Table 1). Sixteen (9.2%) of 174 children had a serum 25(OH)D_3_ concentration below 30 nmol/L, and 89 (51.1%) had a serum 25(OH)D_3_ concentration below 50 nmol/L. At adolescence age, the serum 25(OH)D_3_ concentration decreased (Figure 1). Table 2 shows the mean daily intake of vitamin D per nutrient of three age groups.

**Table 2:** Mean and standard deviation of daily intake of vitamin D per nutrient for three age groups.

Groups	Butter (μg)	Milk (μg)	Fish (μg)	Supplementation (μg)	Total (μg)
Toddlers (1–4 years old)	0.7 ± 0.3	3.2 ± 3.7	0.0 ± 0.1	5.8 ± 4.8	9.7
School-aged (4–12 years old)	0.8 ± 0.5	0.3 ± 1.5	0.1 ± 0.2	3.8 ± 4.6	5.0
Adolescents (>12 years old)	0.6 ± 0.6	0.0 ± 0.2	0.2 ± 0.3	1.8 ± 4.8	2.6

### 3.2. Correlations

Linear regression analysis showed a negative correlation between the age and the total amount of vitamin D obtained from food (*r* = 0.260, *p* = 0.001). There was a positive correlation between the total amount of vitamin D obtained from food and the serum 25(OH)D_3_ concentration (*r* = 0.218, *p* = 0.004). A further positive correlation was detected between milk intake and the serum 25(OH)D_3_ concentration (*r* = 0.186, *p* = 0.014). The other variables (butter, fish or supplements) had no significant effect on the serum 25(OH)D_3_ concentration (Table 3). Pearson correlation analysis showed a significant positive correlation between the total amount of vitamin D obtained from food and milk intake (*r* = 0.951, *p* = 0.000). Therefore, the variable milk intake was excluded in the multiple regression analysis since milk intake had a smaller impact on the serum 25(OH)D_3_ concentration compared to the total amount of vitamin D obtained from food (95% CI 2.1 (0.7–3.5), *p* = 0.004).

If the total amount of vitamin D obtained from food increased with 1 µg, the serum 25(OH)D_3_ concentration increased with 2.1 nmol/L. The *R*-square for the model with the total amount of vitamin D obtained from food was 0.048.

**Table 3:** Results linear regression.

Variables	Linear regression B * (95% CI **); ß, coefficient
Gender	−1.7 (−7.2–3.8), *p* = 0.535; ß = −0.047
Total amount of vitamin D obtained from food	2.1 (0.7–3.5), *p* = 0.004 ***; ß = 0.218
Milk	1.9 (0.4–3.4), *p* = 0.014 ***; ß = 0.186
Butter	4.2 (−0.9–9.3), *p* = 0.106; ß = 0.124
Fish	4.8 (−9.2–18.8), *p* = 0.497; ß = 0.052
Vitamin D supplements	0.3 (−0.3–0.9), *p* = 0.315; ß = 0.077

* Regression coefficient; * 95% confidence interval; *** significant.

### 3.3. Linear Regression

The linear regression analysis showed no statistically significant correlation for the different types of milk and the serum 25(OH)D_3_ concentrations (Table 4). Positive relations can be made between the intake of whole milk and the serum 25(OH)D_3_ concentration (*r* = 0.116, *p* = 0.13). For the residual milk group (toddler milk, soy milk, buttermilk, follow-up milk and yogurt) there was also a positive but not significant relation seen with the serum 25(OH)D_3_ concentrations (*r* = 0.111, *p* = 0.14) (Table 4). Yogurt drinks and semi skimmed milk containing no vitamin D showed small negative relations. None of these findings were significant due to the relatively small sample size. The type of butter was of no influence on the serum 25(OH)D_3_ concentration. Fortified margarine (*r* = −2.2, *p* = 0.66), fortified diet margarine (*r* = 4.5, *p* = 0.13) and non-fortified full fat butter (*r* = 0.4, *p* = 0.94) all showed non-significant relations between their vitamin D content and the serum 25(OH)D_3_. This is surprising considering that the fortified products contain six times more vitamin D compared to the non-fortified full fat butter.

**Table 4:** Results linear regression of different types of milk and serum 25(OH)D_3_ concentration.

Variables	Linear regression B * (95% CI **); ß, coefficient
Yogurt drinks and chocolate milk	−0.6 (−6.7–5.6), *p* = 0.856; ß = −0.014
Semi-skimmed milk	−3.1 (−9.4–3.1), *p* = 0.325; ß = −0.075
Whole milk	7.0 (−2.0–16.0), *p* = 0.127; ß = 0.116
Residual group	5.0 (−1.7–11.7), *p* = 0.143; ß = 0.111

* Regression coefficient; ** 95% confidence interval.

### 3.4. Food or Supplements?

This study investigated the effects of food and vitamin D supplements on the serum 25(OH)D_3_ concentrations between October and April in children aged between 1 and 18 years old. The influence of the total amount of vitamin D obtained from food was examined. In particular, the intake of milk, butter, fish and vitamin D supplements and the different types of milk and butter on the serum 25(OH)D_3_ concentration was closely examined. In our study, vitamin D intake by food showed a stronger relation with serum 25(OH)D_3_ compared to that of intake by supplements.

The results of this epidemiologic pilot study indicate that between October and April, the total amount of vitamin D obtained from food is positive related to serum 25(OH)D_3_ concentration. The type of food with the greatest impact was milk. A positive trend was seen between the intake of whole milk and the residual group (toddler milk, soy milk, buttermilk, follow-on milk and yogurt), and the serum 25(OH)D_3_ concentration. This trend was however not statistically significant.

The effect of food is remarkable; when the total amount of vitamin D obtained from food increased with 1 µg, the serum 25(OH)D_3_ concentration increased with 2.1 nmol/L. The contribution of food can be considered very valuable during winter months.

Due to the limited food fortification with vitamin D and the limited intake of vitamin D supplements in The Netherlands, and considering the different climate zones compared to other countries, it is difficult to compare this research with research from foreign countries. There is generally also very little research on the influence of food and vitamin D supplements on the serum 25(OH)D_3_ concentration in The Netherlands between October and April in pediatric patients.

### 3.5. Intake and Age

The Dutch National Food Consumption Survey investigated the actual intake of vitamin D in a random sample of the Dutch population (*n* = 3819). The advised dietary intake of 2.5 µg vitamin D per day for school aged children and adolescents was found in their cohort with mean intakes of 2.3–3.1 µg/day depending on the age groups. No data was available for children aged below seven years [6]. Our study shows that toddlers have an adequate vitamin D intake but that school-aged children and adolescents do not have an adequate vitamin D intake from dietary sources alone (Table 2). Other studies also show decreasing 25(OH)D_3_ levels with age [11,12,13,14]. When supplementation was added, adequate intake was reached. This could also be seen in the decrease of vitamin D levels related to a particular age group. Furthermore, The Dutch National Food Consumption Survey reported that most of the vitamin D is obtained from basic foods and little vitamin D is obtained from fortified foods or vitamin D supplements [6]. However, our research showed that milk was associated with an increased serum 25(OH)D_3_ concentration. A possible explanation for this difference is that The Dutch National Food Consumption Survey does not contain the same age group tables as our research.

### 3.6. Food Sources

During the winter months 25(OH)D_3_ levels are above zero. It is not likely, however, that a serum 25(OH)D_3_ concentration of 30–50 nmol/L can be reached only with the intake of the foods mentioned above. Other sources for vitamin D should therefore be identified. One explanation could be that we did not include the amount of meat that was eaten as meat which also contains vitamin D_3_. According to the Dutch Food composition database (NEVO tables) [10], fish contains 10–100 times more vitamin D_3_ compared to meat, this made us initially decide not to include the meat products. In a recent study however, a re-analysis of eight other studies also found unidentified sources for serum 25(OH)D_3_ [15]. They measured serum 25(OH)D_3_ and accurately back-calculated basal vitamin D_3_ input. They estimated the basal vitamin D_3_ intake should be 50 µg, irrespective of the source. When the actual vitamin D input from food sources (3–4 µg) and solar sources (8–12 µg) were calculated, a deficit exists of 40 µg between intake and blood concentrations. They, and also other resources, suggested meat sources could be a possible candidate to fill the calculated input gap [15,16]. Since we performed an inventory pilot study, the meat intake should be taken into account in future studies.

The actual amount of vitamin D in milk was not very high but it was an important factor for the final vitamin D levels. This could possibly be due to the use of vitamin D fortification of toddler milk although we did also see this trend in children who did not take toddler milk (Table 4). Ovesen *et al.* [17] also recognized this mechanism, and elucidated it by the content of the metabolite 25-hydroxyvitamin D_3_ in food products which could be more biologically active compared to vitamin D_3_ [17]. Most animal sources contain vitamin D_3_ (which needs to be hydroxylated in the liver), as well as the hydroxylated metabolite, 25(OH)D_3_. The bioactivity of 25(OH)D_3_ in food could be up to five times higher compared to vitamin D_3_. Consensus on this factor is, however, lacking [18]. This activity factor is not included in dietary intake surveys, so true vitamin D intake will typically be underestimated from dietary sources. Additionally, most food composition databases used in different countries (Denmark, France, Germany, Switzerland, Canada or The United States) do not differentiate between vitamin D_2_ and vitamin D_3_. Nor do they give any information about the inclusion or exclusion of the vitamin D metabolites or how the data were obtained as reviewed by Schmid *et al.* [15]. Perhaps this mechanism also plays a role in the butter mechanism. The actual vitamin D content in natural full fat butter was lower compared to margarine. However, the effect on the increase of serum levels by the intake of natural butter was greater than could be expected beforehand. This points out that more factors contribute to the final 25(OH)D_3_ levels in serum. This cannot only be calculated alone based on values mentioned in different databases.

### 3.7. Limitations

A limitation of this study is the use of a food questionnaire. The parents were asked about the food intake over the past week. The mean was calculated to estimate the daily vitamin D intake. A food recall is less reliable compared to that of an actual food diary. To minimize the differences, the same investigator conducted all the food questionnaires. This enabled an even comparison of all the given answers. The parent dependent variable of being able to securely remember the intake could not be taken into account. Existing food composition databases were used to calculate the actual intake of vitamin D_3_ from food. In these databases there was no information on the different types of vitamin D (vitamin D_2_, vitamin D_3_ or 25(OH)D_3_).

Finally, the children in this study all came with a complaint to the pediatrician meaning that they were initially not completely healthy. However, the complaints did not have an impact on vitamin D metabolism so the effect on the serum 25(OH)D_3_ concentration was minimal. 

The results of this study are important because between October and April there is almost no vitamin D synthesis in the skin and many children have a low serum 25(OH)D_3_ concentration during these months. Vitamin D has several important effects in the body, making it important to maintain an adequate serum 25(OH)D_3_ concentration.

## 4. Conclusions 

Between October and April food has an unexpected significant contribution to the serum 25(OH)D_3_ concentration. Supplements with vitamin D were trivial compared to vitamin D rich food sources. Furthermore, the mean serum 25(OH)D_3_ concentration of many children is below the normal range. Due to the important function of vitamin D, it is advisable to recommend nutrition that is rich in vitamin D for children between October and April. Milk is especially important. As the risk for lower 25(OH)D_3_ concentrations increases with age into adolescence, more research is needed on the influence of several factors on the serum 25(OH)D_3_ concentration in adolescents and special attention is needed for this group.
